# Niacin, lutein and zeaxanthin and physical activity have an impact on Charlson comorbidity index using zero-inflated negative binomial regression model: National Health and Nutrition Examination Survey 2013–2014

**DOI:** 10.1186/s12889-019-7906-7

**Published:** 2019-11-28

**Authors:** Hantong Zhao, Changcong Wang, Yingan Pan, Yinpei Guo, Nan Yao, Han Wang, Lina Jin, Bo Li

**Affiliations:** Department of Epidemiology and Biostatistics, School of Public Health, Jilin University, 1163 Xinmin Avenue, Changchun, 130021 People’s Republic of China

**Keywords:** Multimorbidity, Charlson comorbidity index (CCI), Zero-inflated negative binomial (ZINB), Nutrients, Physical activity

## Abstract

**Background:**

Combined with the increasing life expectancy, chronic medical conditions have gradually become the dominant cause of death and disability, and multimorbidity became an increasingly serious public health challenge. However, most existing studies have focused on the coexistence of specific diseases or relatively few diseases. Given one person may have multiple diseases at the same time, we applied Charlson Comorbidity Index (CCI) to systematically evaluate one’s 10-year mortality. In this study, we explored the effects of nutrients and physical activity on CCI using National Health and Nutrition Examination Survey (NHANES) 2013–2014 data.

**Methods:**

The study sample consists of one continuous cycle (2013–2014) of NHANES, and 4386 subjects were included in the study. Nutrients intake was measured by dietary recall, and physical activity was evaluated by the Global Physical Activity Questionnaire respectively. Besides, CCI was the sum of the scores assigned for each medical condition. We utilized zero-inflated negative binomial (ZINB) model to investigate the effects in nutrients intake and physical activity on CCI by adjusting for seven sociodemographic characteristics, smoking and drinking.

**Results:**

Among the 4386 participants, 2018 (68.7%) are Non-Hispanic White, over half participants (78.6%) drink. In count part (CCI ≥ 0), holding other variables constant, the expected change in CCI for a one-unit increase in niacin is 1.621(RR = 1.621, *p* = 0.016), in lutein + zeaxanthin is 0.974 (RR = 0.974, *p* = 0.031), and in sedentary time is 1.035 (RR = 1.035, *p* = 0.005). Moreover, those who do not have vigorous work activity would be more likely to have higher CCI than those who have (RR = 1.275, *P* = 0.045). In logit part (CCI = 0), the log odds of having CCI equals zero would increase by 0.541 and 0.708 for every additional vigorous recreational activity (OR = 0.541, *p* = 0.004) and moderate recreational activity (OR = 0.708, *p* = 0.017) respectively.

**Conclusions:**

Lutein and zeaxanthin intake, vigorous work activity, vigorous recreational activity and moderate recreational activity may be good for one’s health. Rather, increasing niacin intake and sedentary activity may be likely to raise 10-year mortality. Our findings may be significant for preventing diseases and improving health, furthermore, reducing people’s financial burden on healthcare.

## Background

Multimorbidity can be described as one person having multiple diseases at the same time [1]. Multimorbidity has been linked to reduced quality of life, increased mortality and healthcare cost [2]. Combined with the increasing life expectancy, chronic medical conditions have gradually become the dominant cause of death and disability, and multimorbidity became an increasingly serious global public health challenge [3, 4]. As a chronic disease, the death rate of diabetes increased by 32.1% between 2005 and 2015, while the death rate of diabetic nephropathy increased by 39.5% during the same period [5]. Increasing evidences show that diabetic patients will be more likely to suffer from one or more microvascular complications, including cardiovascular diseases (CVD), peripheral neuropathy, blindness, kidney diseases, and so on [6]. Moreover, researches indicated that hypertension, heart failure and diabetes were the most common comorbidities of chronic obstructive pulmonary disease, importantly, the incidence of patients with at least one comorbidity was 84.5% [7]. Furthermore, researchers indicated that there is a correlation between smoking and hepatitis C virus infection in the United States [8]. For infectious diseases, chronic hepatitis B virus (HBV) and hepatitis C virus (HCV) are a major global health burden, which usually results in liver cirrhosis and hepatocellular carcinoma (HCC) [9–11].

However, most existing studies have focused on the coexistence of specific diseases or relatively few diseases, such as diabetes, cardiovascular diseases and cancer, rather than various chronic diseases affecting one person. Moreover, several studies have indicated that social determinants play an important part in pathological changes of the disease and were regarded as the direct cause of some chronic diseases, such as Type 2 diabetes, cardiovascular disease, cancers, infectious diseases and so on [12, 13]. The social determinants includes income, education, occupation, lifestyles, government programs and many other elements that affect the health of individuals [14]. Several studies have shown associations of positive effects of nutrition and lifestyle on hypertension reduction [15, 16]. In addition, recent evidence suggests that smoking and drinking is associated with increased risk of cancers and other chronic diseases [17, 18]. What is more, researchers from large-scale population-based study have indicated that serum folate concentration may be concerned with prostate cancer risk [19]. Furthermore, patterns of dietary, intensity of physical activity and sedentary time play a crucial role in cardiovascular health [20].

Charlson Comorbidity Index (CCI) is a tool used to quantify the total burden of comorbidity and have clinical applications in evaluate one’s 10-year mortality [21–23]. In 1984, CCI was first used to assess the one-year mortality by reviewing hospital charts, which was confirmed in a cohort study of 685 breast cancer patients. Each disease from 19 medical conditions was given a weighting score, and the CCI was the sum of all the scores [23]. The standard regression model usually applies to a normal distribution. Although Poisson regression is suitable for modeling counts, it assumes the variance and mean of the distribution are equal. Besides, negative binomial (NB) model allows for the variance to exceed the mean because of its dispersion parameter, but it unable to model too many zeros counts well [24]. Zero-inflated negative binomial (ZINB) model is appropriate for modeling over-dispersed and zero-inflated data [25, 26], which assumes the zeros come from two sub-classes. Specifically, one population constitutes observations that might contain zero counts (at risk class) because of sampling while another population is composed of observations that always contain zeros (not at risk class). In general, logistic regression (logit link) is employed to distinguish structural zeros from sampling zeros and positive counts. Besides, NB or Poisson model is used to model the counts that might contain zeros. Furthermore, we can estimate odds ratios (OR) and risk ratios (RR) from the logit part and count part of the model, respectively [24].

The aim of this study was to explore the relative contribution of nutrients and physical activity to 10-year mortality, which will provide valuable information for public health professionals, providers, and policymakers. And improve quality of life, reduce mortality as well. Given one person may have various diseases at the same time, we applied CCI to systematically evaluate one’s 10-year mortality. Different from other studies, we explored the effects of nutrients and physical activity on one’s 10-year mortality using National Health and Nutrition Examination Survey (NHANES) 2013–2014 data by ZINB model.

## Methods

### Study population

The study sample consists of one continuous cycle (2013–2014) of NHANES, which used a stratified multistage probability cluster design to be representative of the civilian, noninstitutionalized U.S. population, conducted by the National Center for Health Statistics, under the Centers for Disease Control and Prevention [27, 28]. A total of 10,175 individuals participated in NHANES during 2013–2014, but 5769 adults who were 20 years of age or older were restricted to our study. We excluded participants who were pregnant or lactating (*n* = 104). Besides, adults with caloric intakes of < 500 kcal or > 5000 kcal per day were excluded (*n* = 60). Then, we excluded 1219 participants due to missing covariate information, leaving 4386 eligible subjects for the study. Study protocols for NHANES were approved by the National Center for Health Statistics ethnics review board (https://www.cdc.gov/nchs/nhanes/irba98.htm). All the participants signed the informed consent before participating in the study. The dataset is available at https://wwwn.cdc.gov/nchs/nhanes/continuousnhanes/default.aspx?BeginYear=2013.

### Assessment of sociodemographic and lifestyle characteristics

The study included a number of covariates evaluated as potential confounding factors, such as age (20–39; 40–59; 60 and above); gender (male, female); race/ethnicity (Hispanic, Non-Hispanic White, Non-Hispanic Black, Other Race); educational attainment (less than high school; high school, including general equivalent diploma; college or higher); income ($0–$24,999, $25,000–$54,999, $55,000–$74,999, $75,000 or above); smoking (yes, no); drinking (yes, no).

In addition, physical activity was also incorporated into the paper. The Physical Activity Questionnaire is based on the Global Physical Activity Questionnaire [29], and the questions were asked using the Computer-Assisted Personal Interview software. The main outcomes of physical activity were defined using the following questions: (1) Sedentary activity: “How much time do you usually spend sitting on a typical day?”; (2) Vigorous work activity: “Does your work involve vigorous-intensity activity that causes large increases in breathing or heart rate like carrying or lifting heavy loads, digging or construction work for at least 10 minutes continuously?” (yes, no); (3) Moderate work activity: “Does your work involve moderate-intensity activity that causes small increases in breathing or heart rate such as brisk walking or carrying light loads for at least 10 minutes continuously?” (yes, no); (4) Moderate recreational physical activity: “Do you do any moderate-intensity sports, fitness, or recreational activity that cause a small increase in breathing or heart rate such as brisk walking, bicycling, swimming, or volleyball for at least 10 minutes continuously?” (yes, no); (5) Vigorous recreational physical activity: “Do you do any vigorous-intensity sports, fitness, or recreational activity that cause large increases in breathing or heart rate like running or basketball for at least 10 minutes continuously?” (yes, no).

### Assessment of dietary nutrients

NHANES subjects were asked for two averaged 24-h recalls of dietary intakes using the USDA’s Automated Multiple-Pass method [30]. The first dietary recall was conducted by trained dietary interviewers, they gathered detailed information on all the foods and beverages participants consumed in the past 24 h, and a second recall was administered by telephone 3 to 10 days later. The total dietary nutrients taken through food and beverages are averaged over two days, nonetheless, if one did not complete the second dietary interview, only the first interview was used. Dietary supplements were also evaluated by the information on the number of days they were taken and the amount that were taken in the past 30 days. What is more, the average intake for 30 days is the total amount of supplement. Furthermore, total dietary nutrients intake was evaluated by foods and supplements.

### Response variable

Forty-nine chronic diseases (including infectious diseases, like HBV and HCV) were included in the study, which were self-reported using the NHANES questionnaire. Chronic HBV and chronic HCV are considered as a global public health problem, which usually lead to liver cirrhosis and HCC [9–11], so they were included in this study. Diagnosed diseases were defined by positive responses to one or more questions such as: “Have you ever been told that you have the illness?” and “Are you now taking some pills to control your disease?”. People who do not report whether they suffer from a certain disease are considered healthy, which means the disease condition is assigned a value of zero. And we did this for the following reasons. Firstly, those who “do not know” the answer are most likely to have never been diagnosed by patients, because they do not have the symptoms of certain disease. Thus, we consider them do not suffer from such disease. What is more, the occurrence of chronic diseases is a slow progress. And it takes a long time from the change of body to the appearance of symptoms, which brings us to keep a conservative estimate. Meanwhile, this also avoid reducing the sample size by excluding the participants with missing values, so as to improve the representativeness and reliability of the study. The comprehensive health status of a participant was measured by the CCI [31], which is shown in Additional file 1. Derived from adding up the scores of forty-nine diseases, CCI evaluates a participant’s severity of comorbidity and the mortality in 10 years. The 10-year mortality (Y) is calculated as follows: Y = e ^ (CCI ∗ 0.9), which can be obtained at website https://fpnotebook.com/prevent/Exam/ChrlsnCmrbdtyIndx.htm#fpnContent-panel-id_6.

### Statistical analyses

**Fig. 1:**
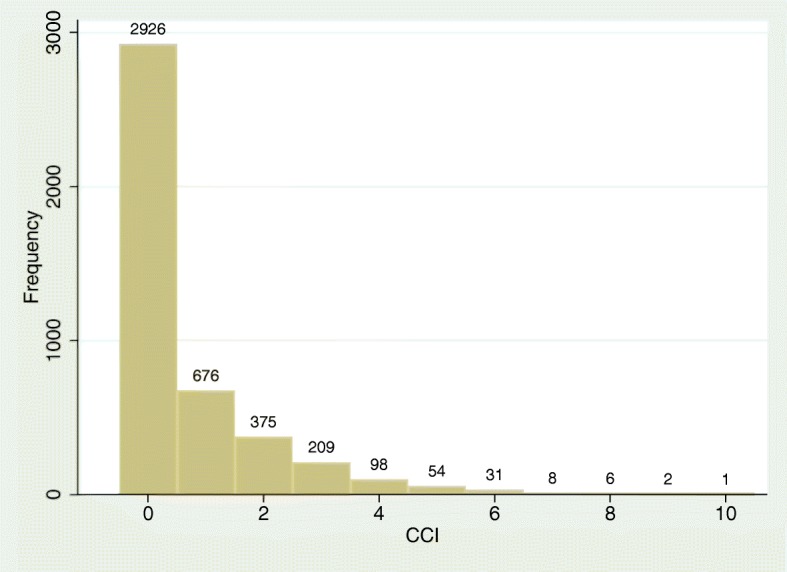
Distribution Carlson comorbidity index among respondents aged 20 years and above, NHANES 2013–2014 (*n* = 4386)

Figure 1 demonstrates that the distribution of CCI is non-normal count data and the variance is greater than the mean, which could be deemed as over-dispersed data. Simultaneously, there are too many zero counts presented in Fig. 1, and NB model unable to model CCI well. CCI was assumed as an over-dispersed and zero-inflated count variable, so we utilized ZINB model to appropriately model it [24, 32]. The model examined the association of the exposure variables, in this case dietary nutrients and physical activity, with the outcome variable (CCI) in two parts. Specifically, the logit part models CCI equals zero (structural zero), and count part models CCI equals to zero (sampling zero) and positive counts. Seven covariates including age, gender, race, education, income, smoking and drinking were adjusted for both in the logit part and count part.

All analyses account for clusters pseudo-strata, pseudo-sampling units and participant weights to accommodate the complex sampling of the data, which were performed by using Stata/SE, version 15.1 statistical software. Two-sided *p* < 0.05 was considered significant for statistical inferences.

## Results

Among the 4386 participants, 2018 (68.7%) are Non-Hispanic White, 946(13.8%) are Hispanic, 863 (10.5%) are Non-Hispanic Black, and 559 (7.0%) are other races (including multi-racial). In addition, 2396 (55.2%) are GED/AA degree. In the respect of drinking, over half participants (78.6%) drink. On average, the intake of niacin is 37.1 ± 1.34 mg, and sedentary activity is 7.1 ± 0.10 h. Other information related to background characterises, nutrient intake and physical activity are shown in Tables 1 and 2. The frequency distribution of CCI is shown in Fig. 1, and there are 66.7% of the respondents with CCI equals zero.

**Table 1:** Background characteristics of respondents aged 20 years and above, NHANES 2013–2014 (*n* = 4386)

	Study Participants
Variable	*n* (%^a^)
Age	
20–39	1456 (34.9)
40–59	1501 (37.2)
60 and above	1429 (27.9)
Gender	
Male	2154 (49.6)
Female	2232 (50.4)
Race/ethnicity	
Hispanic	946 (13.8)
Non-Hispanic White	2018 (68.7)
Non-Hispanic Black	863 (10.5)
Other Races (Including Multi-Racial)	559 (7.0)
Educational attainment	
Less than High school	842 (13.6)
GED/AA degree	2396 (55.2)
College and higher	1148 (31.2)
Income	
0–24,999	1343 (22.5)
25,000–54,999	1275 (27.1)
55,000–74,999	470 (12.0)
75,000 and above	1298 (38.4)
Smoking	
Yes	1942 (43.8)
No	2444 (56.2)
Drinking	
Yes	3219 (78.6)
No	1167 (21.4)

^a^All data are weighted to be nationally representative

**Table 2:** Nutrients intake and physical activity of respondents aged 20 years and above, NHANES 2013–2014 (*n* = 4386)

	Study Participants
Variable	Mean (SE) * or *n* (%*)
Protein (gm)	82.7 (0.93)
Carbohydrate (gm)	245.3 (1.80)
Dietary fiber (gm)	17.0 (0.27)
Total fat (gm)	80.4 (0.76)
Vitamin A (mcg)	634.5 (15.22)
Thiamin (Vitamin B1) (mg)	7.5 (1.24)
Riboflavin (Vitamin B2) (mg)	4.6 (0.31)
Niacin (mg)	37.1 (1.34)
Vitamin B6 (mg)	7.4 (1.96)
Folate (mcg)	526.8 (9.63)
Vitamin B12 (mcg)	78.3 (6.60)
Vitamin C (mg)	178.7 (9.00)
Vitamin K (mcg)	133.8 (6.00)
Vitamin D (D2 + D3) (mcg)	18.1 (0.77)
Vitamin E (mg)	10.1 (0.22)
Calcium (gm)	1.1 (0.02)
Phosphorus (gm)	1.4 (0.02)
Magnesium (mg)	331.9 (5.5)
Iron (mg)	17.9 (0.34)
Zinc (mg)	15.5 (0.30)
Copper (mg)	1.5 (0.03)
Sodium (gm)	3.5 (0.03)
Potassium (gm)	2.7 (0.04)
Selenium (mcg)	132.9 (1.65)
Water (kg)	2.9 (0.05)
Cholesterol (mg)	292.5 (4.46)
Lycopene (mg)	5.1 (0.11)
Lutein + zeaxanthin (mg)	1.8 (0.06)
Alpha-carotene (mcg)	419.3 (22.9)
Beta-carotene (mg)	2.3 (0.10)
Beta-cryptoxanthin (mcg)	84.9 (3.11)
Total choline (mg)	336.2 (4.84)
Hours sedentary activity	7.1 (0.10)
Vigorous work activity	
Yes	847 (20.1)
No	3539 (79.9)
Moderate work activity	
Yes	1516 (36.9)
No	2870 (63.1)
Vigorous recreational activity	
Yes	1017 (25.3)
No	3369 (74.7)
Moderate recreational activity	
Yes	1870 (45.6)
No	2516 (54.4)

*All data are weighted to be nationally representative

The results of count part were presented in Table 3. In count part (CCI ≥ 0), holding other variables constant, the expected change in CCI for a one-unit increase in niacin is 1.621 (RR = 1.62, *p* = 0.016), the expected change in CCI for a one-unit increase in lutein + zeaxanthin is 0.974 (RR = 0.974, *p* = 0.031), and the expected change in CCI for a one-unit increase in sedentary time is 1.035 (RR = 1.035, *p* = 0.005). Moreover, a non-vigorous work activity has an expected RR of 1.275 higher than a vigorous work activity holding other variables constant (RR = 1.275, *P* = 0.045).

**Table 3:** Predictors of Carlson comorbidity index among respondents aged 20 years and above, NHANES 2013–2014^a^ (*n* = 4386)

	Count part		Logit part	
Variable	RR (95% CI)	*p*-Value	OR (95% CI)	*p*-Value
Protein (kg)	0.171 (0.01,3.03)	0.211	36.039 (0.27,4817.45)	0.140
Carbohydrate (kg)	0.462 (0.19,1.11)	0.079	0.562 (0.10,3.11)	0.484
Dietary fiber (gm)	0.00013 (6.19e-9,2.65)	0.074	0.010 (2.92e-10,510,936.20)	0.600
Total fat (kg)	0.223 (0.03,1.66)	0.132	0.167 (0.0023,12.03)	0.386
Vitamin A (mg)	0.934 (0.76,1.14)	0.479	1.059 (0.79,1.41)	0.677
Thiamin (Vitamin B1) (gm)	0.820 (0.42,1.60)	0.538	0.089 (< 0.01,169.97)	0.506
Riboflavin (Vitamin B2) (gm)	25.700 (0.01,46,630.03)	0.371	25.482 (< 0.01,150,994.70)	0.439
Niacin (gm)	1.621 (1.11,2.37)	0.016	0.445 (0.08,2.54)	0.337
Vitamin B6 (gm)	15.134 (0.24,958.15)	0.183	3.590 (< 0.01,58,454.27)	0.783
Folate (mg)	1.085 (0.94,1.25)	0.240	1.021 (0.74,1.41)	0.890
Vitamin B12 (mg)	0.988 (0.90,1.08)	0.786	0.925 (0.75,1.14)	0.437
Vitamin C (gm)	1.111 (0.93,1.33)	0.239	0.860 (0.55,1,34)	0.479
Vitamin K (mg)	1.007 (0.99,1.02)	0.231	0.929 (0.85,1.02)	0.106
Vitamin D (D2 + D3) (mg)	2.242 (0.72,7.01)	0.152	0.383 (0.01,16.37)	0.594
Vitamin E (mg)	0.998 (< 0.01,14,191.52)	0.764	3897.15 (< 0.01,173,249,744,453.59)	0.333
Calcium (gm)	1.072 (0.97,1.19)	0.170	1.206 (0.99,1.47)	0.065
Phosphorus (gm)	0.933 (0.77,1.13)	0.448	1.274 (0.93,1.74)	0.118
Magnesium (gm)	1.004 (0.67,1.49)	0.984	1.374 (0.73,2.59)	0.303
Iron (gm)	2.711 (0.07,100.16)	0.565	0.332 (< 0.01,4216.24)	0.807
Zinc (gm)	7.067 (0.09,529.69)	0.350	3.406 (< 0.01,3,953,057.94)	0.854
Copper (mg)	1.018 (1.10e-15,1.69e+ 30)	0.482	1.042 (2.37e-43,3.66e+ 78)	0.537
Sodium (gm)	0.959 (0.91,1.01)	0.123	1.049 (0.93,1.18)	0.419
Potassium (gm)	0.924 (0.83,1.03)	0.157	1.045 (0.93,1.17)	0.432
Selenium (mg)	0.862 (0.47,1.59)	0.612	4.182 (0.30,59.44)	0.268
Water (kg)	0.983 (0.93,1.04)	0.559	1.052 (0.96,1.16)	0.273
Cholesterol (gm)	0.994 (0.67,1.47)	0.973	1.527 (0.52,4.48)	0.415
Lycopene (mg)	0.992 (0.98,1.00)	0.140	1.008 (0.99,1.03)	0.479
Lutein+zeaxanthin (mg)	0.974 (0.95,0.99)	0.031	0.972 (0.90,1.06)	0.477
Alpha-carotene (mg)	0.945 (0.85,1.05)	0.253	0.879 (0.62,1.25)	0.450
Beta-carotene (mg)	0.987 (0.955,1.02)	0.411	0.992 (0.94,1.05)	0.785
Beta-cryptoxanthin (mg)	0.863 (0.71,1.05)	0.133	0.602 (0.25,1.46)	0.240
Total choline (gm)	0.828 (0.50,1.38)	0.442	1.639 (0.61,4.43)	0.306
Hours sedentary activity	1.035 (1.01,1.06)	0.005	1.001 (0.96,1.05)	0.948
Vigorous work activity				
Yes	Reference		Reference	
No	1.275 (1.01,1.62)	0.045	1.501 (0.98,2.30)	0.060
Moderate work activity				
Yes	Reference		Reference	
No	0.984 (0.87,1.12)	0.788	0.842 (0.52,1.36)	0.457
Vigorous recreational activity				
Yes	Reference		Reference	
No	0.977 (0.78,1.22)	0.828	0.541 (0.37,0.79)	0.004
Moderate recreational activity				
Yes	Reference		Reference	
No	1.030 (0.89,1.20)	0.680	0.708 (0.54,0.93)	0.017

^a^All data are weighted to be nationally representative

Table 3 also contains weighted ORs from logit part of ZINB model. In logit part (CCI = 0), the log odds of having CCI equals zero would increase by 0.541 for every additional vigorous recreational activity (OR = 0.541, *p* = 0.004), and the log odds of having CCI equals zero would increase by 0.708 for every additional moderate recreational activity (OR = 0.708, *p* = 0.017).

After adjusting for the background characteristics (age, gender, race, education, income, smoking and drinking), we have the following principal findings. With the increasing intakes of niacin, participants were more likely to suffer from an increase in CCI. However, lutein and zeaxanthin might have beneficial impact on individuals’ health and decrease mortality. In the respect of physical activity, the death rate among people who have higher sedentary time and do not have vigorous work activity will be more likely to increase. In addition, persons do not have vigorous or moderate recreational activity have the risk of death compared with those who have.

## Discussion

### Niacin

In our study, we found the higher the total niacin intake, the higher the risk of death. Although there are limited studies that directly look at the association between niacin and CCI, the relationship between niacin and certain disease plays the role of indirect explanation. Similar to our findings, Park et al. [33] reported that there is a positive correlation between niacin intake and basal cell carcinoma risk, the authors also indicated that the higher the total niacin intake, the higher the risk of melanoma risk in men, but not in women. This also provides new ideas for our future research, because we did not analyze gender differences. Niacin promote the synthesis of sex hormones, and affect the development of melanocytes, and higher estrogen exposure increases the risk of skin melanoma [34, 35]. Although high doses of niacin is used as a cholesterol-lowering drug clinically, side effects on the body are also obvious, such as causing vasodilation, skin flushing, headache and hypotension [36, 37]. However, the intakes of niacin always accompanied with other B group vitamins or other nutrients, and this might mask the opposite relationship. In addition, in this study, niacin intake is 37.1 ± 1.34 mg averagely presented in Table 2, which marginally exceeds recommended allowances of nutrients [38]. And it is easily accumulated in the body because of the properties of fat-soluble, which might be partially reasons of adverse relationship between niacin and mortality. So, the results may be due to chance. Thus, future studies are need in this area.

### Lutein and zeaxanthin

Our study showed a negative correlation between lutein and zeaxanthin intake and 10-year mortality. Although it’s hard to find studies that directly look at the correlation between lutein and zeaxanthin and CCI, the relationship between lutein and zeaxanthin and certain disease plays the role of indirect explanation. In this way, our result is in agreement with plenty of earlier studies. One recent research suggested that the more lutein and zeaxanthin consumed, the lower risk of colorectal cancer [39]. In addition, Heinen et al. [40] indicated that lutein and zeaxanthin also plays a crucial role in skin cancer. Moreover, researchers demonstrated that lutein and zeaxanthin may reduce the risk of cancer types especially breast and lung cancers, they also indicated its function of suppressing heart disease and stroke [41]. Its impact on preventing and controlling diseases might attribute to the following mechanisms. Firstly, acting as anti-oxidant, they reduce oxidative damage indirectly by absorbing light [42, 43]. Secondly, early studies indicated that lutein and zeaxanthin may protect against inflammation by inhibiting the increase of oxidation-induced cytokines and upregulating the expression of inflammation-related genes [44]. Besides, as anti-oxidants, zeaxanthin effectively remove water-soluble and lipid-soluble peroxyl radicals [45]. Furthermore, animal studies suggested that lutein and zeaxanthin could reduce the adverse effects of inflammatory cytokines and low density lipoprotein on blood vessels [46, 47]. These reports all support our finding.

### Nutrients without statistically significance

Our study did not find any associations between other nutrients intake and CCI. Consistent with our report, researchers of a cohort study reported that vitamin K intake was not associated with mortality [48], and Vinceti et al. [49] indicated that increasing selenium intake through diet and supplement may not prevent cancer. Besides, a systematic review and meta-analysis of prospective cohort studies demonstrated that dietary choline intake have no effect on incident CVD, and authors also find the association between dietary choline intake and CVD mortality was not statistically significant [50]. Table 3 shows the 95% confidence interval (CI) of OR and RR values for dietary fiber, vitamin E, copper, etc. is very large. This may be attributed to the insufficient sample size, leading to the instability of results. However, some of our results differed from several others. Authors of a prior study of middle and older-aged adults aged 40 and older years (NHANES, 1988–1994) found the relationship between vitamin D status and cardiometabolic mortality [51]. Our results may differ from others due to age difference, and dietary recall is less accurate than a biological sample. In addition, in contrast to a cohort study of Spanish graduates’ findings of correlations between vitamin C intake and cardiovascular mortality [52], we found no relationship between vitamin C intake and mortality, which may be attributed to ethnic factors. Furthermore, the differences in our study results and those of other studies might be partially due to the potential confounding factors and measurement differences. Furthermore, statistical model plays a crucial role as well.

### Sedentary time and physical activity

We concluded that individuals who have higher sedentary time and do not have vigorous work activity will be more likely to have higher rate of death. And people who do not have vigorous recreational activity or moderate recreational activity have the risk of death. Previous studies have reported that there is a negative correlation between health status and sedentary time, which is associated with chronic diseases and overall mortality [53, 54]. For adults and the elderly, inactivity in leisure time and high television time increase the incidence of chronic diseases [55]. Moreover, a research from NHANES 2009–2012 pointed out that increasing time spent on physical activity will lead to lower risk of chronic diseases [56]. However, some researchers suggested that high activity level could weaken the increased risk associated with excessive sedentary time [57]. Importantly, this provide us with a novel idea that our study could consider the interaction between physical activity and sedentary time in further studies.

### Strengths and limitations

There are several strengths of this work. Firstly, including a representative sample with a large size, and NHANES employs thorough quality assurance procedures, which ensure the validity of the data. Secondly, wide ranges of potential confounders such as demographic characteristics and behavior elements were controlled to provide a better estimate of the association between nutrient intake and physical activity and CCI. Thirdly, we applied CCI to systematically evaluate one’s 10-year mortality, for one may have various diseases at the same time. Lastly, this study is the first to explore the associations between nutrients intake and physical activity and CCI by using ZINB model. However, several limitations of this study should be noted. Firstly, some of the participants were excluded due to the missing data, and we have a conservative assignment for CCI. Secondly, a dietary recall is less accurate than a urine sample, which may result in a false estimate of nutrients intake. Furthermore, we cannot infer a causal relationship because this was a cross-sectional study. Lastly, the count part includes sampling zero, and the logit part includes structural zero, but ZINB model cannot distinguish the causes of their generation well and may result in biased estimates.

## Conclusion

In conclusion, our findings suggest the associations between nutrient intakes and physical activity and CCI. Lutein and zeaxanthin intake, vigorous work activity, vigorous recreational activity and moderate recreational activity may be good for one’s health. Rather, increasing niacin intake and sedentary activity may be more likely to raise 10-year mortality. Given the high prevalence of chronic diseases in recent years, and our results, it is crucial to continue research of nutrient intake and physical activity and CCI. Our findings may be extremely significant for preventing diseases and improving health, furthermore, reducing people’s financial burden on healthcare and improving the life quality.

## Supplementary information

**Additional file 1.** The sores of forty-nine diseases, NHANES 2013-2014 (n= 4386).

## Data Availability

The datasets generated and/or analyzed during the current study are publicly available from the NHANES website (https://wwwn.cdc.gov/nchs/nhanes/continuousnhanes/default.aspx?BeginYear=2013.

## Abbreviations

CCI: Charlson Comorbidity Index
CI: Confidence interval
CVD: Cardiovascular disease
HBV: Hepatitis B virus
HCC: Hepatocellular carcinoma
HCV: Hepatitis C virus
NB: Negative binomial
NHANES: National Health and Nutrition Examination Survey
OR: Odds ratio
RR: Rate ratio
ZINB: Zero-inflated negative binomial
